# A comprehensive look at transcription factor gene expression changes in colorectal adenomas

**DOI:** 10.1186/1471-2407-14-46

**Published:** 2014-01-29

**Authors:** Janine Vonlanthen, Michal J Okoniewski, Mirco Menigatti, Elisa Cattaneo, Daniela Pellegrini-Ochsner, Ritva Haider, Josef Jiricny, Teresa Staiano, Federico Buffoli, Giancarlo Marra

**Affiliations:** 1 Institute of Molecular Cancer Research, University of Zurich, Winterthurerstrasse 190, Zurich 8051, Switzerland; 2 Functional Genomics Center, University of Zurich and ETH Zurich, Zurich, Switzerland; 3 Institute of Pathology, Triemli Hospital Zurich, Zurich, Switzerland; 4 Endoscopy and Gastroenterology Unit, Hospital of Cremona, Cremona, Italy

**Keywords:** Transcription factors, Gene expression, Colorectal adenomas, DACH1

## Abstract

**Background:**

Biological processes are controlled by transcription networks. Expression changes of transcription factor (TF) genes in precancerous lesions are therefore crucial events in tumorigenesis. Our aim was to obtain a comprehensive picture of these changes in colorectal adenomas.

**Methods:**

Using a 3-pronged selection procedure, we analyzed transcriptomic data on 34 human tissue samples (17 adenomas and paired samples of normal mucosa, all collected with ethics committee approval and written, informed patient consent) to identify TFs with highly significant tumor-associated gene expression changes whose potential roles in colorectal tumorigenesis have been under-researched. Microarray data were subjected to stringent statistical analysis of TF expression in tumor vs. normal tissues, MetaCore-mediated identification of TF networks displaying enrichment for genes that were differentially expressed in tumors, and a novel quantitative analysis of the publications examining the TF genes’ roles in colorectal tumorigenesis.

**Results:**

The 261 TF genes identified with this procedure included *DACH1,* which plays essential roles in the proper proliferation and differentiation of retinal and leg precursor cell populations in *Drosophila melanogaster.* Its possible roles in colorectal tumorigenesis are completely unknown, but it was found to be markedly overexpressed (mRNA and protein) in all colorectal adenomas and in most colorectal carcinomas. However, DACH1 expression was absent in some carcinomas, most of which were DNA mismatch-repair deficient. When networks were built using the set of TF genes identified by all three selection procedures, as well as the entire set of transcriptomic changes in adenomas, five hub genes (*TGFB1*, *BIRC5, MYB*, *NR3C1*, and *TERT*) where identified as putatively crucial components of the adenomatous transformation process.

**Conclusion:**

The transcription-regulating network of colorectal adenomas (compared with that of normal colorectal mucosa) is characterized by significantly altered expression of over 250 TF genes, many of which have never been investigated in relation to colorectal tumorigenesis.

## Background

Colorectal adenomas are benign tumors of the large intestinal epithelium. They are found in roughly one third of asymptomatic adults who undergo colonoscopy before the age of 50. Endoscopic removal of these lesions is associated with high rates of recurrence (up to 60% at three years, depending on the size, number, histological features, and degree of dysplasia [1]). In addition, it has been estimated that 15% of adenomas measuring 1 cm or more become carcinomas within 10 years of their detection [2].

Adenomatous transformation of normal colorectal mucosa is associated with profound changes in the tissue’s gene expression profile [3]. These changes are caused by epigenetic and/or genetic events that “reprogram” the regulation of gene transcription [4]. An early—and probably fundamental—event in this reprogramming process involves qualitative, quantitative, and spatial subversion of the Wnt signaling pathway, the physiological regulator of epithelial homeostasis [5]. Almost invariably, it stems from mutations in genes encoding Wnt pathway components (*APC*, *adenomatous polyposis coli*, in most cases), which lead to the accumulation of β-catenin in both the cytoplasm and nucleus. In the latter compartment, it interacts with DNA-binding proteins of the T-cell factor/lymphoid-enhancer factor family, transforming them from transcriptional repressors into transcriptional activators.

The abnormal activation of Wnt signaling can affect the expression of numerous genes involved in epithelial homeostasis, including the oncogenic transcription factor (TF)-encoding gene *MYC*. It is one of the genes most frequently found to be overexpressed in intestinal adenomas and carcinomas (and many other tumors as well) [6,7]. Genes directly targeted by MYC have been identified in various tumors [8,9], but more recent studies suggest that this oncogene might be a “universal amplifier” with effects on most of the cell’s actively expressed genes. This phenomenon might account for the broad spectrum of effects ascribed to this oncogene in normal and tumor cells [10,11].

However, while MYC undoubtedly plays a central role in tumors that overexpress it, the adenomatous phenotype is likely to be underpinned by transcription networks in which the expression of numerous TFs is altered. These networks are characterized by cross-regulation and redundant regulation of component TFs and TF-gene binding that occurs over a wide range of DNA occupancy levels [12]. Understanding how the concentration of a given TF in a neoplastic tissue differs from that in its normal tissue counterpart is therefore of paramount importance to elucidate the tumorigenic process.

Gene expression studies can reveal potentially important factors in colorectal tumorigenesis by pinpointing genes with markedly up- or downregulated expression levels in early precancerous lesions [3,13,14]. For this reason, we attempted in the present study to comprehensively characterize the TF gene expression changes that occur in colorectal adenomas. Many of the numerous changes we identified involve TF genes that have not been previously linked to colorectal tumorigenesis. One of these, *DACH1*, consistently displayed marked upregulation in the colorectal adenomas we examined, and it was subjected to further investigation in a series of neoplasms representing different types and stages of colorectal tumor progression.

## Methods

### Microarray data

We analyzed previously collected [13] gene expression data on 17 pedunculated colorectal adenomas and 17 peritumoral samples of normal mucosa (> 2 cm from the adenoma). The pathologic features of the tumor series are summarized in Additional file 1: Table S1. Human colorectal tissues were prospectively collected from patients undergoing colonoscopy in the *Istituti Ospitalieri* of Cremona, Italy. The approval of the ethics committee of this institution was obtained, and tissues were used in accordance with the Declaration of Helsinki. Each donor provided written informed consent to sample collection, data analysis, and publication of the findings. Detailed descriptions of RNA extraction method and the Affymetrix Exon 1.0 microarray analysis are available in the report of our original study [13]. Raw transcriptomic data have been deposited in GEO (accession number GSE21962).

### Selection of TF genes

**Figure 1:**
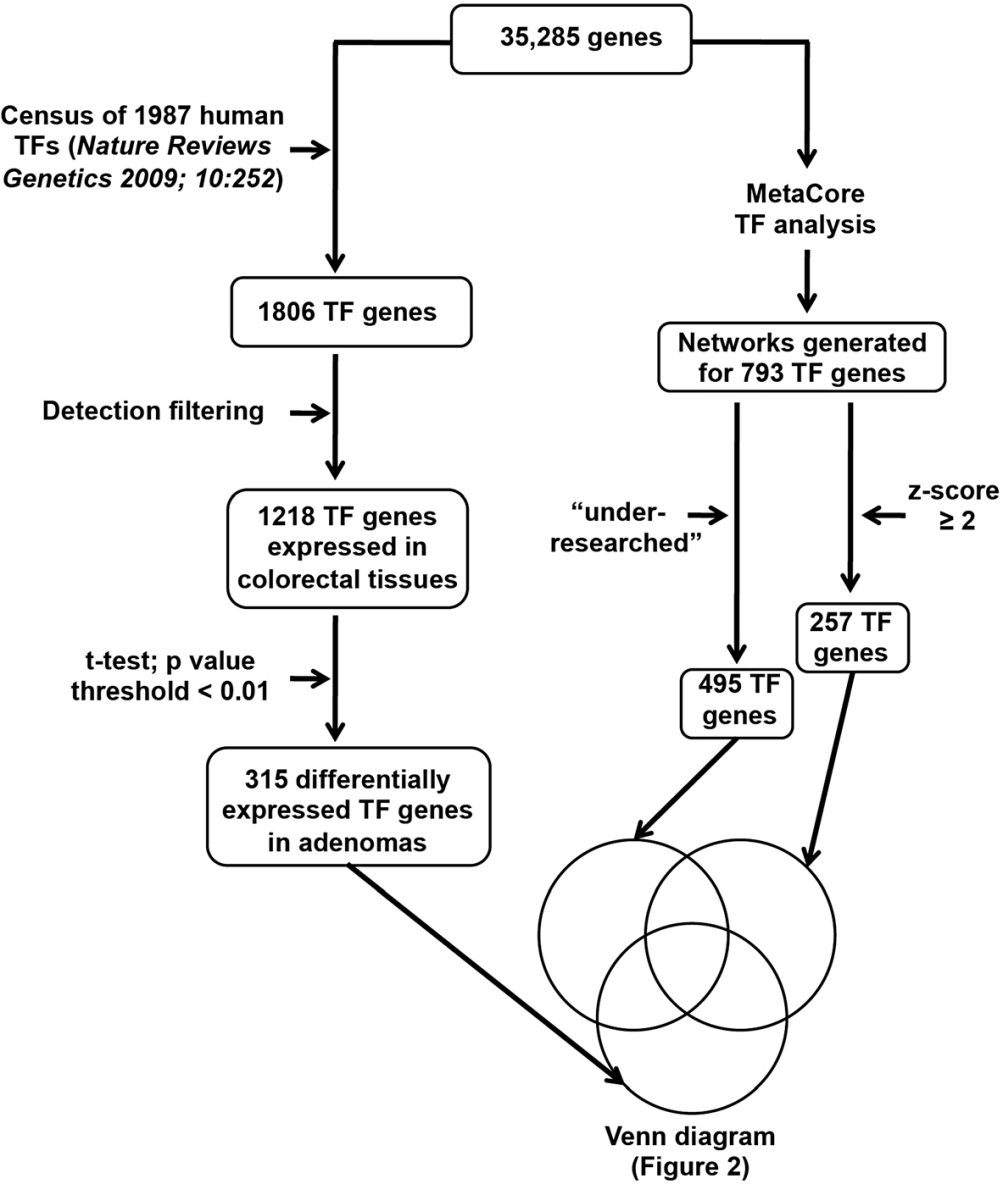
**Three-pronged procedure used to select 261 transcription factor (TF) genes with probable but relatively unexplored roles in colorectal tumorigenesis.** The initial data set included 35,285 genes (including 23,768 annotated protein-encoding genes) represented on the Affymetrix Exon 1.0 microarray used to analyze 17 colorectal adenomas and corresponding specimens of normal mucosa. Left prong: Selection of 315 genes that encode TFs, are expressed in normal and/or adenomatous colorectal mucosa, and display significantly up- or downregulated transcription in adenomas. Middle and right prongs: MetaCore TF analysis identified 793 TF genes whose interaction networks were enriched for genes that were significantly up- or downregulated in adenomas. This list was then filtered to identify those with z scores of ≥2 (n = 257) and those with NormPDIs of >0 (n = 495) (see Methods section for details).

A three-pronged selection procedure (Figure 1) was used to identify TFs likely to play important but unsuspected roles in colorectal tumorigenesis. The starting point was a list of 35,285 genes, i.e., the 23,768 protein-encoding genes examined in the original study [13] plus 11,517 non-protein-encoding genes.

First (Figure 1, left prong), these genes were screened against a census of human TFs published in 2009 by Vaquerizas *et al*. [15]. This manually curated compilation contains 1987 sequence-specific DNA-binding TF genes, each with information on its function, genomic organization, and evolutionary conservation. Most were identified with the Ensemble Genome Browser [16], but 27 are probable TF genes from other sources, such as Gene Ontology [GO] or TRANScription FACtor [TRANSFAC] database [17]. One thousand eight hundred six of the 1987 TF genes in the census were also found in our original data set. These genes were selected on the basis of gene-level Brainarray summaries [18] of the Exon 1.0 microarray data, so exon-level and splicing information were not taken into account. A detection filter was then applied to select TF genes likely to be expressed in either normal or adenomatous colorectal tissues. Candidates were thus excluded unless their expression values exceeded an arbitrarily defined cut-off of 5.8 (log_2_ scale) in ≥ 50% of the samples in one or both of the tissue groups (adenomas, normal mucosa). The 1218 TF genes selected with this step are listed in Additional file 2: Table S2. This list was then further reduced to include only those TF genes that had exhibited significantly up- or downregulated expression in the adenomas vs. normal mucosa (TF genes in bold face in Additional file 2: Table S2). For this final selection, a p value threshold of < 0.01 in a paired two-tailed t test was chosen. Unadjusted p values were used for the ranking, which is not influenced by multiple testing correction [19].

The second and third prongs of the selection procedure (Figure 1, middle and right-hand columns) began with analysis of TF genes in the original data set with commercially available MetaCore™ software (version 6.14, build 61508) from GeneGo, Inc. In MetaCore, each gene is assigned to a network of related genes (e.g., a TF gene is included in a network of genes that it likely regulates). Network size varies widely: some contain less than 10 genes, others (like that of the transcription factor SP1), well over 2000. The MetaCore TF analysis used the hypergeometric test to select TF genes regulating networks enriched in genes that had displayed significant differential expression in our adenomas, as compared with normal mucosa. The results are expressed in terms of a z-score, which reflects the deviation stretch from the mean of a normally distributed population, and a p value, which is inversely correlated with the significance of the TF network (Additional file 3: Table S3). We set a relaxed significance threshold (a t-test p value of 0.2 and an absolute logarithmic fold change of 0.2) to select TF networks with enough significant elements to allow efficient calculation of enrichment. The significance of a given TF gene network in the context of the selected genes, measured by hypergeometric test, is described by its p value and additionally by the z-score of network enrichment. The 793 TF genes whose networks were enriched in genes displaying significant differential expression in adenomas (Figure 1) are listed in Additional file 4: Table S4, where those with z-scores > 2 are reported in bold-face type.

MetaCore is based on a curated database of human protein-protein and protein-DNA interactions, transcription factors, signaling and metabolic pathways, diseases and toxicity, and the effects of bioactive molecules. It is constructed and edited manually by GeneGo scientists on the basis of data from full-text articles published in relevant journals (https://portal.genego.com). The size of a gene network therefore depends on the data (and therefore the number of publications) available on a given gene. In GeneGo, TF significance (measured by the parameters described above) is related to network size. Therefore, genes that have been researched more intensively and are therefore well-represented in published reports might be reported as more significant than those that have been less thoroughly investigated. In other words, higher connectivity might be partly rooted in investigative biases.

The third prong of our selection procedure (Figure 1) was designed to correct for such biases by identifying TFs that are under-represented in scientific publications dealing with colorectal tumors. For each TF gene identified by the Metacore analysis, we manually reviewed the GeneCard (http://www.genecards.org) links to research articles dealing with the gene indexed in PubMed (as well as Novoseek, HGNC, Entrez Gene, UniProB, PharmaGKB and/or GAD) and recorded the number of articles that also dealt with colorectal tumors (*actual* publications). Correlation between the number of *actual* publications and the z-score of each TF gene was assessed with a scatter plot, and a trend line was drawn to identify the *expected* number of publications for each TF (Additional file 5: Figure S1). The trend line was obtained by multiplying the z-score for each TF by the slope value (142 in this case, with the fixed intercept = 0). The correlation was fairly strong (=0.4) for such heterogeneous data, so the linear approximation appeared to be justified. By subtracting the *actual* number from the *expected* number of publications calculated for each TF, the *difference in publications* (DP) was obtained. The normalized DP (NormDP) was then calculated [i.e., NormDP = (*actual* - *expected* publication number)/*expected* publication number], which correlates with the distance to the trend line. Higher NormDPs reflect larger discrepancies between the expected and actual numbers of publications and are therefore associated with TFs whose possible links to colorectal tumorigenesis have been relatively “under-researched.” The TF genes with a NormDP > 0 were therefore termed "under-researched" (the 495 TF genes in red colour in Additional file 4: Table S4). Their importance and number of connections in the Metacore database may be underestimated owing to their limited presence in the literature.

The TF gene sets generated by the three selection procedures were compared and their intersections represented in a Venn diagram (see *Results* and *Discussion* sections). Hierarchical clustering analysis of the microarray data was carried out using heatmap.2 function from the gplots library (CRAN repository at http://cran.rproject.org/web/packages/gplots/index.html) with Pearson correlation as a distance function and Ward agglomeration method for clustering.

The TF gene expression perturbations found in our adenoma series were also compared with those reported in advanced colorectal tumors. For this purpose, we applied the same TF gene selection procedure to the Exon 1.0 microarray-based, gene expression data reported by Maglietta et al. [14] (raw data available in Array Express E-MTAB-829) relative to 13 colorectal *carcinomas* and paired samples of normal mucosa.

### Immunohistochemistry

Immunostaining was used to assess DACH1 protein expression patterns in 20 archival, formalin-fixed, paraffin-embedded colorectal adenomas, 80 sporadic colorectal cancers, and the normal mucosa adjacent to these latter lesions. The cancers represented different stages and histologic grades (Additional file 6: Table S5). Forty were classified as mismatch repair (MMR)-proficient and 40 as MMR-deficient based on immunostaining for the protein encoded by the MMR gene *MLH1,* whose lack of expression in sporadic cancer is caused by CpG island hypermethylation at its promoter [20]. MLH1 protein expression in a cancer tissue is usually uniformly strong (indicating MMR proficiency) or completely absent (MMR deficiency) [20].

In brief, 4-μm sections of each cancer were mounted on glass slides coated with organosilane (DakoCytomation), deparaffinized, and rehydrated. Antigen retrieval was accomplished by heating the sections in a pressure cooker at 120°C for 2 min in 10 mM citrate-buffered solution (pH 6.0). DAKO peroxidase-blocking reagent and goat serum were used sequentially to suppress nonspecific staining due to endogenous peroxidase activity and nonspecific antibody binding, respectively. Sections were then incubated overnight at 4°C with the primary antibody (mouse monoclonal anti-MLH1 antibody [BD, no. 551091, 1:200 dilution] or rabbit polyclonal anti-DACH1 antibody [Sigma, no. HPA012672, 1:400 dilution]). The sections were washed, and appropriate secondary antibodies conjugated to peroxidase-labeled polymer (DAKO EnVision + kit) were applied for 30 min at RT. Finally, the sections were incubated with 3,3’diaminobenzidine chromogen solution (DAKO) to develop the peroxidase activity and then counterstained with hematoxylin.

DACH1 immunostaining patterns proved to be complex and were evaluated as follows. The extension of staining in each cancer specimen (i.e., the percentage of tumor cells displaying any degree of staining) was rated as absent (no stained cells); limited (≤ 35% cells); moderate (36%–69%); or extensive (70%–100%). As for immunostaining intensity, scores were first assigned to various areas of the cancer (1 = weak; 2 = moderate; 3 = strong). The highest score assigned anywhere in the cancer specimen was then added to the score that was most representative of the specimen. The sum was an intensity score ranging from 2 to 6. The Fisher exact test was used to examine the significance of associations between extension or intensity DACH1 staining score and various characteristics of the cancers (MMR status, TNM stage, and histologic grade).

The specificity of the DACH1 antibody we used was verified in immunostaining experiments performed as described above on sections of formalin-fixed, paraffin-embedded pellets made from colon cancer cell lines with different *DACH1* gene expression levels.

### Evaluation of DACH1 promoter methylation status in colorectal cancers

Using the QIAamp DNA FFPE Tissue kit (Qiagen, no. 56404), we extracted DNA from 18 of the 80 cancers described above. DACH1 expression in these cancers was marked and ubiquitous in 6, patchy in 6, and completely lost in 6 (see examples in the *Results* section), and each of these 3 groups included 3 tumors that were MMR-proficient and 3 that were MMR-deficient. Sodium bisulfite conversion of genomic DNA was performed as previously described [21], and the resulting DNA was subjected to combined bisulfite restriction analysis (COBRA) to determine the methylation status of two CpG islands located respectively upstream the transcription start site (CpG I) and in the first intron (CpG II) of the *DACH1* gene. Amplifications were carried out with FastStart Taq DNA Polymerase (Roche, Basel, Switzerland) with the following primers: CpG I: 5’-GTAGTAGTAGAAGAGAAGTAGATGA-3’ (sense) and 5’- ACCCAAATTATCCAACCAAAAACTC-3’ (antisense); CpG II: 5’-GGGTGAGGGTTTIGTTGGGA-3’ (sense) and 5’-CCCTCCCCTCIACTAACTTC-3’ (antisense). The amplified products were digested with the *Taq*^*α*^*I* restriction enzyme (New England Biolabs, Beverly, MA, USA) and subjected to 2% agarose gel electrophoresis and ethidium bromide staining.

## Results

To isolate *bona fide* TFs from our original set of 35,285 genes, we screened it against the census of 1987 human TFs compiled by Vaquerizas *et al*. [15]. As shown in Figure 1 (left-hand prong), 1806 of the 1987 TF genes were identified among those in our original set, but only 1218 of these were significantly expressed in either normal colorectal mucosa or in colorectal adenomas or in both (see *Methods*). The expression levels of these 1218 TF genes in the normal and neoplastic tissue groups are illustrated in a hierarchical clustering analysis of the 34 tissue samples (Additional file 7: Figure S2). As shown in Figure 1 (and detailed in Additional file 2: Table S2), 315 of the 1218 TF genes were found to be significantly over- or under-expressed in adenomas relative to normal mucosa (t test: p < 0.01).

Parallel MetaCore analysis of the original gene set identified 793 TF genes whose interaction networks were enriched for genes displaying significant differential expression in adenomas, as compared with normal mucosa samples (Additional file 4: Table S4). This list, which was generated with the relatively relaxed criteria described in the *Methods* section, was then filtered (Figure 1, right-hand prong) to select the TF genes most likely to be involved in adenomatous transformation of the colorectal epithelium. The result was a list of 257 TF genes with z-scores ≥ 2 in the hypergeometric enrichment test, reflecting gene expression changes in adenomas amounting to at least 2 standard deviations from the mean expression change.

In parallel, the MetaCore list of 793 TF genes was filtered to identify those whose possible role in colorectal tumorigenesis has been relatively under-researched (Figure 1, middle prong), as defined by the NormDP (see *Methods*). This analysis pinpointed 495 of the 793 TF genes with fewer than expected publications on their involvement in colorectal tumorigenesis (i.e., NormDPs of >0; Additional file 4: Table S4).

**Figure 2:**
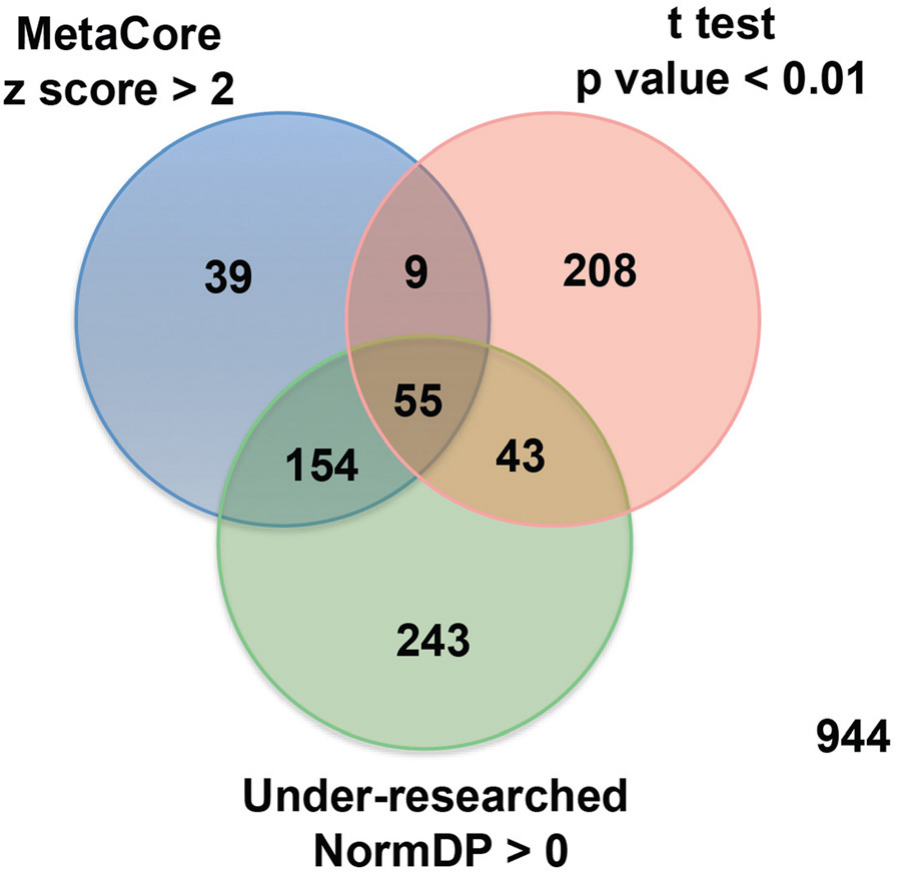
**Venn diagram showing intersection of TF gene sets selected in Figure **1**.** One thousand sixty seven TF genes were identified in at least one of the three selection procedures described in Figure 1. Two hundred sixty-one TF genes were identified in two of the selection procedures and 55 were selected in all three procedures.

**Figure 3:**
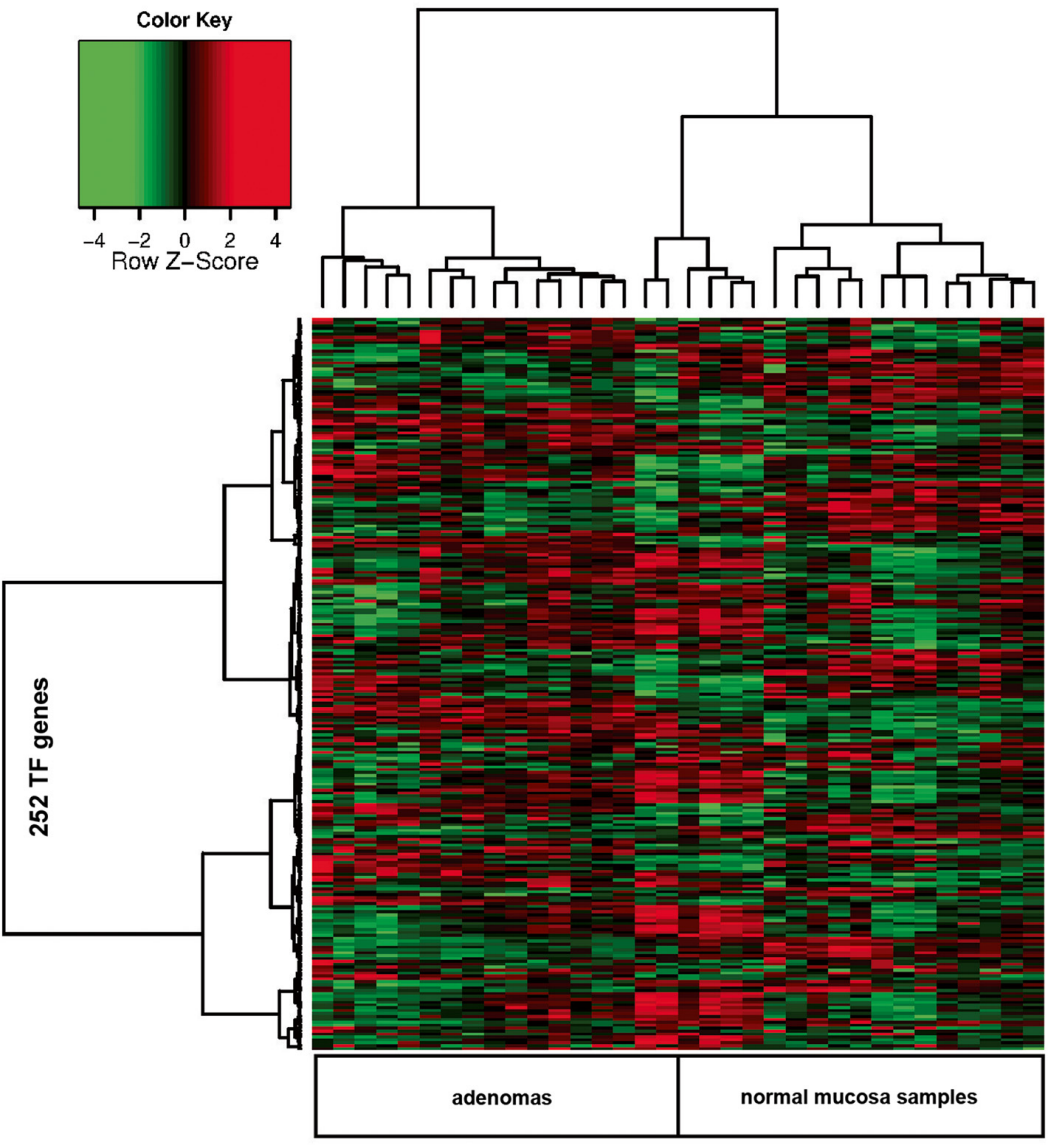
**Hierarchical clustering analysis of colorectal tissue samples based on the TF genes found in two of the three sets shown in Figure **1**.** (Pearson correlation, Ward distance). The 34 tissue samples represented on the *x*-axis include 17 normal mucosal samples and 17 adenomas. Each transcript probe set plotted on the *y*-axis is color-coded to reflect expression levels of the TF genes relative to their median expression levels across the entire tissue-sample set (red: high; green: low). Two hundred fifty-two of the 261 TF genes listed in Additional file 8: Table S8 are reported here: the other 9 (i.e., the last 9 in Additional file 8: Table S8) were not among the 35,285 genes represented on the Affymetrix Exon 1.0 microarray platform, but they were considered in networks generated with the MetaCore TF analysis.

Figure 2 shows the intersections of the three TF gene sets obtained with the procedures described above. Two hundred sixty one were identified with at least two selection procedures (Additional file 8: Table S8). Hierarchical clustering analysis of the 34 tissue samples based on the expression levels of these TF genes showed clear separation of the adenomas and normal mucosa samples (Figure 3). The sub-clusters of adenomas and normal samples seen in Figure 3 showed no correlation with the known clinical and pathologic features of the tissues (Additional file 1: Table S1), which is not particularly surprising given the relatively small number of samples analyzed.

We then used two different approaches to identify TF genes listed in Additional file 8: Table S8 that might be candidates for subsequent validation studies as drivers of colorectal transformation. First, using manual inspection of the list, we selected the TF genes with the following characteristics: marked upregulation in adenomas (i.e., top upregulated genes in Additional file 8: Table S8) and *no* actual publications on the possible roles in colorectal tumorigenesis (regardless of whether research had been published on their involvement in other types of tumorigenesis). Upregulated TF genes were chosen since they were also more likely to represent potential biomarkers of adenomatous transformation.

**Figure 4:**
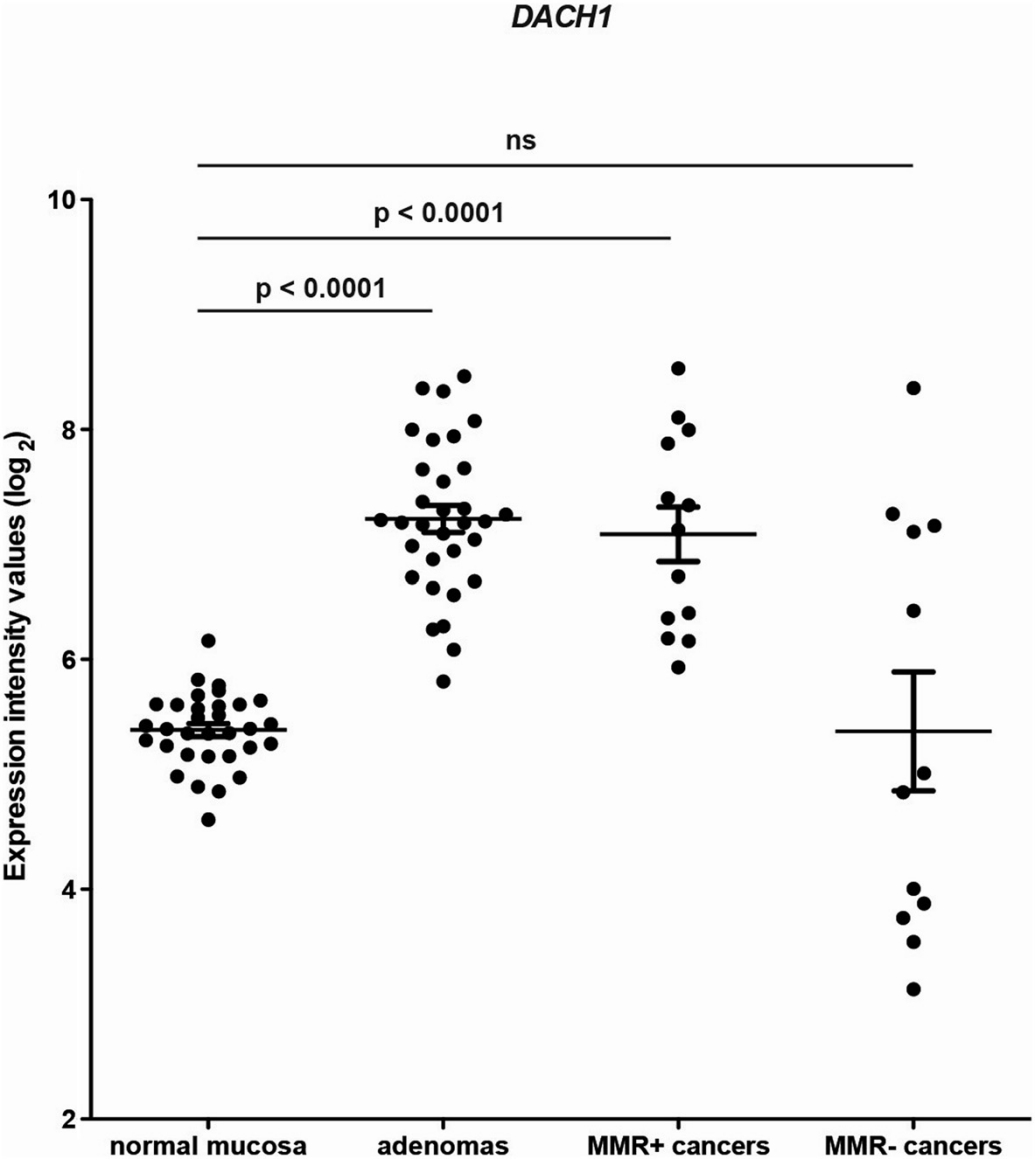
***DACH1 *mRNA expression in normal colorectal mucosa, colorectal adenomas, and mismatch repair (MMR)-deficient and -proficient colorectal cancers.** Scatter plot of normalized log_2_ expression intensity values for *DACH1* (Affymetrix U133 Plus 2.0 array analysis) in the 4 tissue groups analyzed in our previous study [3]. Means and standard errors are represented by horizontal lines and t-bars, respectively.

One of the genes that met these criteria was *DACH1*. Microarray data from a previous study by our group [3] had indicated that its expression is also upregulated in most colorectal cancers, although significantly reduced mRNA levels were observed in some of the cancers tested, all of which were MMR-deficient (Figure 4). This observation prompted us to conduct immunohistochemistry experiments to investigate DACH1 protein expression in colorectal adenomas and in colorectal cancers of different stages, histologic grades, and MMR status (40 MMR + and 40 MMR-, Additional file 6: Table S5).

**Figure 5:**
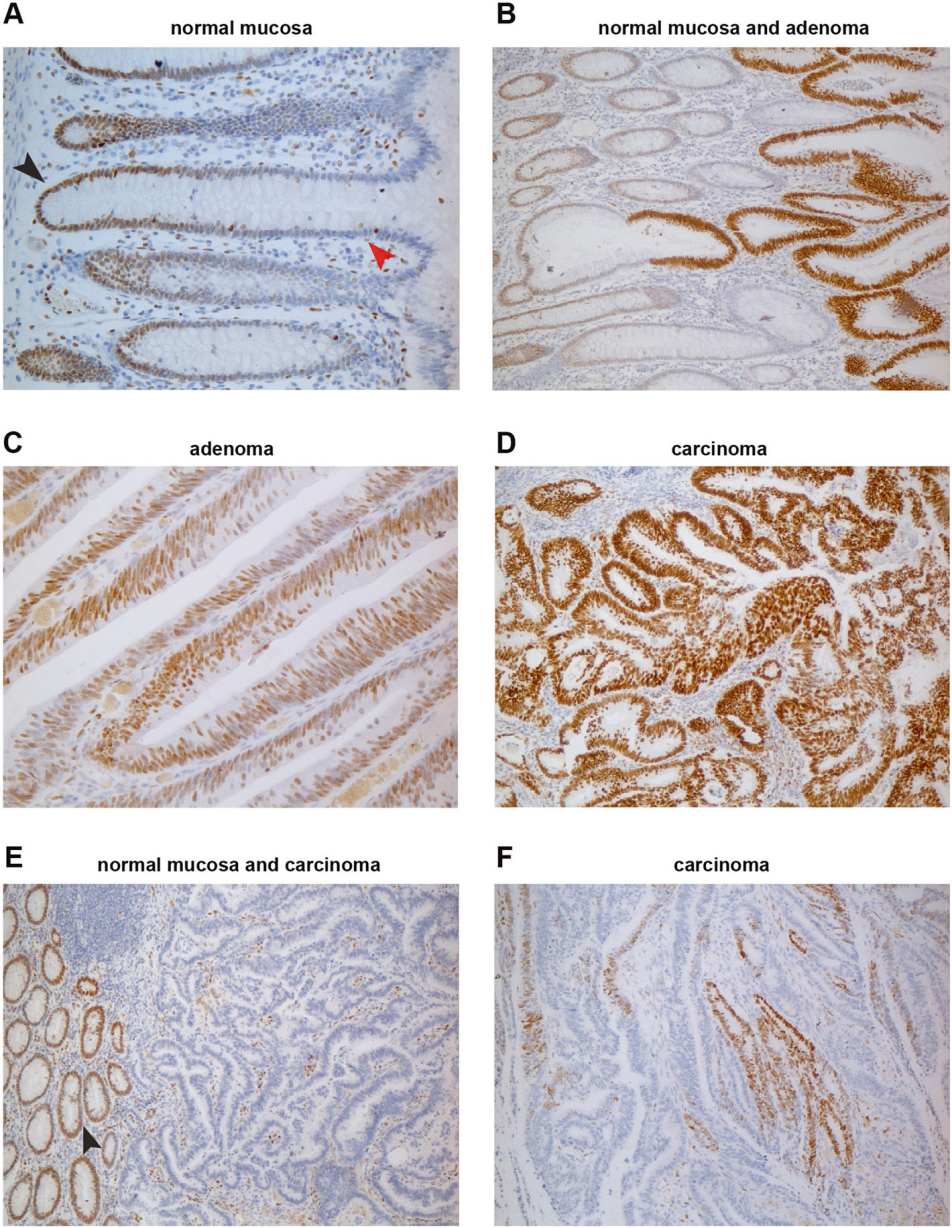
**Immunohistochemical staining for DACH1 protein in normal and neoplastic colon. (A)** In normal mucosa, DACH1 expression is present in the nuclei of proliferating cells in the lower portion of the epithelial crypts (black arrowhead) and completely absent in the differentiated cells in the upper crypts (red arrowhead). **(B)** High-level DACH1 expression is seen in rapidly proliferating cells of adenomatous glands taking over normal crypts. Abundant expression is also seen in most cells of a colorectal adenoma **(C)** and a colorectal carcinoma **(D)**. In another colorectal cancer **(E)**, DACH1 expression is absent in neoplastic glands, although proliferating cells in the normal mucosa and in the tumoral stroma are positive. **(F)** A third colorectal cancer with patchy staining for DACH1.

The DACH1 antibody used for these studies displayed excellent specificity, as shown by Additional file 9: Figure S3. Immunostaining of normal mucosa revealed high nuclear expression of DACH1, which was confined mainly to the proliferating cells in the lower half of colorectal crypts (Figure 5A). Nuclear expression was also invariably strong in the adenomas we tested, but in this case it was almost ubiquitous (Figure 5B and C). As for the cancers, three different staining patterns emerged: marked and ubiquitous DACH1 expression resembling that seen in adenomas (Figure 5D); complete loss of expression throughout the lesion (Figure 5E); and patches of variable-intensity staining interspersed with areas of absent expression (Figure 5F). The latter two patterns were significantly more frequent in MMR- cancers (30/40 vs. 11/40 of those that were MMR+). Fisher’s exact tests showed that DACH1 expression in MMR- cancers was significantly more likely to be partially/completely lost (staining extension: <70% of cells; p = 0.00016) or relatively weak (intensity scores of <5) (p = 0.054) than that observed in MMR+ cancers. DACH1 staining intensity scores were also significantly lower in poorly differentiated (G3) cancers (p = 0.019 vs. G2 cancers), which were (as expected [20]) significantly associated with MMR deficiency (P = 0.0019). DACH1 staining patterns did not appear to be related to TNM stages, although this finding needs to be confirmed in larger groups of MMR+ and MMR- cancers.

**Figure 6:**
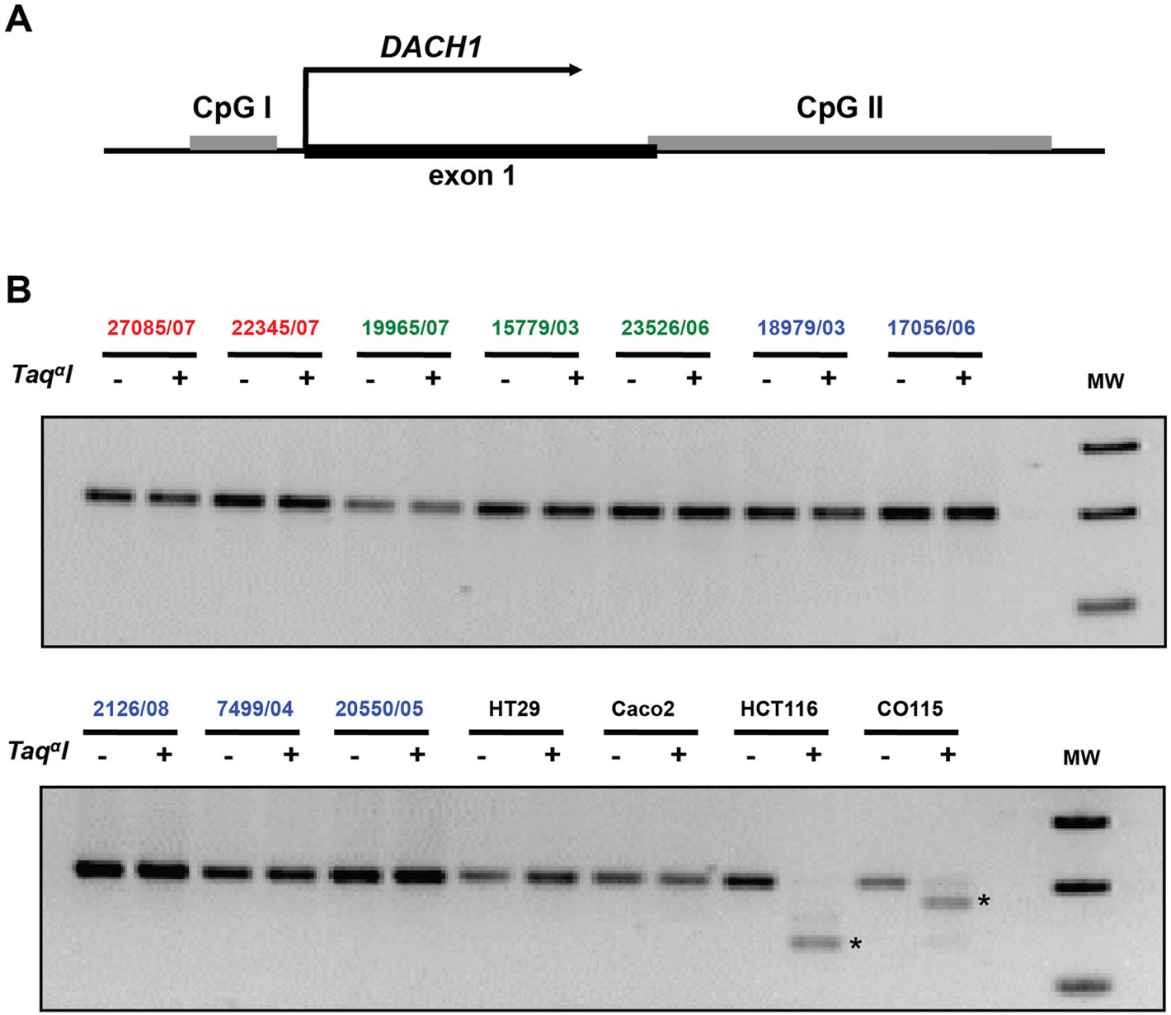
**Methylation analysis of the CpG island in the *DACH1 *promoter. (A)**: Schematic depiction of the CpG islands located respectively 5’ upstream from the *DACH1* transcription start site (CpG I) and in the first intron of the *DACH1* gene (CpG II). **(B)**: Examples of CpG I COBRA analysis in colorectal cancers with intense (red), patchy (green), or no (blue) DACH1 protein immunostaining and in 4 colon cancer cell lines characterized by low (HT29 and Caco2) or very low (HCT116 and CO115) *DACH1* expression (based on microarray-documented *DACH1* mRNA expression levels - see also Additional file 9). Asterisks indicate *Taq*^*α*^*I*-digested DNA fragments representing methylated alleles; slower-migrating fragments correspond to undigested, unmethylated DNA. MW, molecular weight; bp, base pair.

Because our MMR- cancers showed loss of gene expression due to epigenetic silencing of the MMR gene *MLH1*, we wondered whether their diminished DACH1 expression might be caused by methylation at the *DACH1* promoter. The COBRA experiments we performed failed to confirm this hypothesis. The CpG island located in the *DACH1* promoter (CpG I in Figure 6A, primers in *Methods*) was not found to be methylated in any of the 18 cancers we tested (samples from each DACH1 staining pattern group are shown in Figure 6B). Hypermethylation at this site may occur *in vitro,* however, as shown for the colon cancer cell lines HCT116 and CO115 (Figure 6B). Similar results were obtained with the COBRA analysis of a different CpG island located in the first intron of the *DACH1* gene (CpG II in Figure 6A).

**Figure 7:**
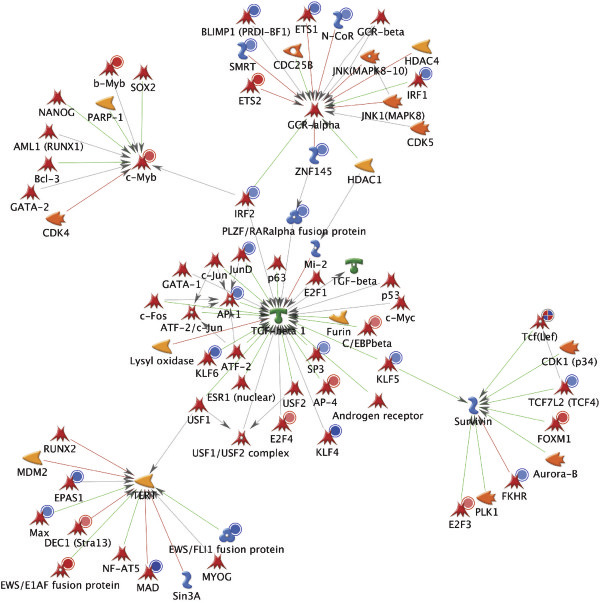
**Organic (hub-centric) layout of the most significant network identified by MetaCore.** The network includes 27 of the 55 TF genes found in all three sets depicted in Figure 2.

The second approach we used involved the identification of genes that might represent important hubs in the transcriptional network of adenomas (as opposed to the one operating in the normal mucosa). To this end, we uploaded the 55 significant TF genes identified by all three selection procedures (Figure 2) into the MetaCore database and ran a comparative analysis of their networks. The most promising network included the following five target genes: *TGF-beta 1* (*TGFB1*), *TERT*, *Survivin* (*BIRC5*), *c-Myb* (*MYB*), and *GCR-alpha* (*NR3C1*) (see Figure 7, and Additional file 10: Figure S4 and Additional file 11: Figure S5). This network was characterized by a p value of 3.43e-64 and 75 target genes, including 27 “seeds”, i.e., TF genes. These findings will be discussed in the next section.

Finally, we compared the perturbations of TF gene expression documented in our colorectal adenomas with those reported by Maglietta et al. [14] in 13 colorectal *carcinomas* and paired normal mucosa samples. These latter tissue pairs were a subset of the 17 analyzed by Maglietta et al. They were selected because they had all been processed in the same laboratory [14]. As shown in Additional file 12: Figure S6), a substantial proportion of TF genes whose expression was dysregulated in the carcinomas were also dysregulated in our adenomas (46% using the *t* test based-approach of the left prong of our selection procedure, 57% using the MetaCore-based approach of the right prong [Figure 1]). The TF genes identified in colorectal carcinomas with these two approaches are reported in Additional file 13: Table S6 and Additional file 14: Table S7).

## Discussion

The aim of this study was to identify TF genes with probable roles in the early stages of colorectal tumorigenesis, especially those whose roles in this setting have been less extensively investigated. The list we compiled contained 261 TF genes, including one, *DACH1*, which appeared particularly interesting. It was invariably overexpressed in the preinvasive stage of colorectal tumorigenesis (i.e., adenomas) and frequently upregulated in colorectal cancers as well. However, it was found to be silenced in certain colorectal cancers, especially those that were MMR-.

To our knowledge, this is the first attempt to comprehensively characterize the TF gene transcriptome of human colorectal adenomatous polyps, although several studies have been published on the overall transcriptional profile of colorectal tumors (GEO database [22] and our previous reports [3,13,23]). Vaquerizas *et al*. studied TF gene expression changes in 32 healthy human tissue types, but, surprisingly, the colorectum was not included.

The focus of our study was the adenomatous colorectal polyp (as compared with corresponding samples of normal colorectal mucosa), and genes were considered to be expressed in these tissues only if they had expression levels of ≥5.8 (log scale). This cutoff, which was chosen on the basis of our previous observations and the recommendations of the microarray manufacturer (Affymetrix), is less stringent than the one used by Vaquerizas *et al.*[15]. We deliberately chose a more relaxed cutoff to maximize our chances of identifying all TFs involved in colorectal carcinogenesis, even those with low-level expression. This is important because TF mRNA and TF proteins are less stable than those of other classes of genes [24], and TF protein levels span over four orders of magnitude [12]. The number of TF genes that met our criterion for expression in normal or adenomatous colorectal tissue (or both)—1218—was thus markedly higher than those reported by Vaquerizas *et al*. in normal tissues of other organ systems, which ranged from 150 to 300 [15]. The U133 Plus 2.0 array used by these investigators is also less sensitive than the Affymetrix exon array platform we used [13]. In spite of these differences, however, in both studies, TF genes represented ~7% of all genes classified as “expressed” genes in most of the tissues examined.

The three-pronged selection procedure we used to identify TF genes involved in colorectal tumorigenesis generated a final list of 261 candidates (Additional file 8: Table S8). At the time of our analysis, only 15% of these genes had been implicated (putatively or otherwise) in colorectal tumorigenesis in more than 10 publications, including a few like *MYC* and *TP53*, whose links to this process are well-established. And for 102 (39%) of the candidate genes, our literature search revealed no data at all on their possible roles in colorectal tumors.

To extract meaningful biological information from this list, we initially focused on the TF genes displaying the most markedly *upregulated* expression in colorectal adenomas together with the lowest publication scores. One of the top genes in this subgroup was *DACH1*, a human homolog of the *Drosophila melanogaster* TF gene *dachshund*, which is essential for proper proliferation and differentiation of retinal and leg precursor cell populations in these flies [25-27]. *DACH1* appears to regulate the transcription of several human genes involved in proliferation (e.g., *CDKN1B, CCND1, JUN*, and *TGFb*) [28-32]. Furthermore, our immunohistochemistry studies revealed abundant DACH1 expression in the nuclei of epithelial cells in the lower half of normal colorectal crypts (Figure 5A), where proliferation predominates over differentiation. Therefore, the staining pattern strongly associates *DACH1* expression with cell proliferation and/or commitment to cell differentiation. It has also recently found to be highly expressed in cycling intestinal stem cells from mice [33].

In line with these findings, the expression of *DACH1* mRNA and protein was significantly increased in tumor lesions (Figures 4 and 5C/D), which are extensively populated by proliferating cells. However, it does not appear to be indispensable for cancer-cell proliferation and cancer progression since some of the colorectal cancers we examined were characterized by complete or partial loss of DACH1 protein expression (Figure 5E and F). These losses showed no correlation with the TNM stages of the cancers, but they were significantly more frequent in tumors that were poorly differentiated and/or MMR deficient.

The mismatch repair defect was the result of epigenetic silencing of the *MLH1* gene, and a similar phenomenon might have been responsible for the loss of DACH1 expression in some cancers. However, COBRA revealed no evidence of cytosine hypermethylation at the CpG islands investigated (Figure 6A) in any of the colorectal cancers we examined (although analysis of colon cancer cell lines indicated that this phenomenon can occur *in vitro*) (Figure 6B). The loss of DACH1 in certain cancers might stem from cytosine hypermethylation at other possible regulatory regions of the *DACH1* locus or from other types of epigenetic changes at this site. Additional work is needed to explore these possibilities. In any case, histone modifications are likely to play some role in the silencing of this gene. *DACH1* is one of the developmental TF genes whose chromatin in mouse embryonic stem cells is bivalent, i.e., it harbors permissive as well as repressive histone marks (Figure 2 in [34]). This epigenetic conformation, which facilitates the gene’s ability to switch rapidly between transcriptionally active and inactive states, might account for the staining patterns shown in Figure 5.

Altered *DACH1* expression has already been reported in other human tumors. In a study of lung cancers based on whole-genome sequencing, *DACH1* emerged as a biologically significant target of mutation (loss-of-function alterations in particular) [35], and its homozygous deletion has been reported in some glioblastoma multiformes [36]. More recently, DACH1 has been reported to inhibit the growth of lung adenocarcinoma cells through its binding to TP53 [37]. DACH1’s putative tumor suppressive function has also been documented in studies of breast, prostate, and uterine cancers (reviewed in [38]), where its expression was found to be frequently downregulated. In contrast, upregulated expression has been reported in advanced ovarian cancers [39] and in CD15+ myeloid progenitor cells harboring the t(9;11) translocation [40], and there is some evidence that it exerts oncogenic effects in t(9;11) acute myeloid leukemia [41].

In light of these findings, the transcription-regulating roles of *DACH1* in somatic tissues—and their implications for tumorigenesis in a given tissue— might be expected to vary widely. The anti-*DACH1* antibody used in our study is highly reliable for exploring this question, but it has been commercially available only recently. Immunostaining patterns in extracolonic tissues and tumors obtained with older antibodies might therefore need to be re-examined.

A second approach used in the analysis of the TF genes listed in Additional file 8: Table S8 involved the identification of hub genes in networks that could be built with the selected TF genes. Like many other proteins, TFs interact with the products of other genes. Interaction networks are very useful to better understand the functional significance of gene expression changes. Each TF influences the expression of several genes, producing changes in the levels of mRNA and, in many cases, also in the levels of the corresponding proteins. Thus, it is important to know the expression level of the TF gene itself, but also that of the other genes in its network(s).

**Figure 8:**
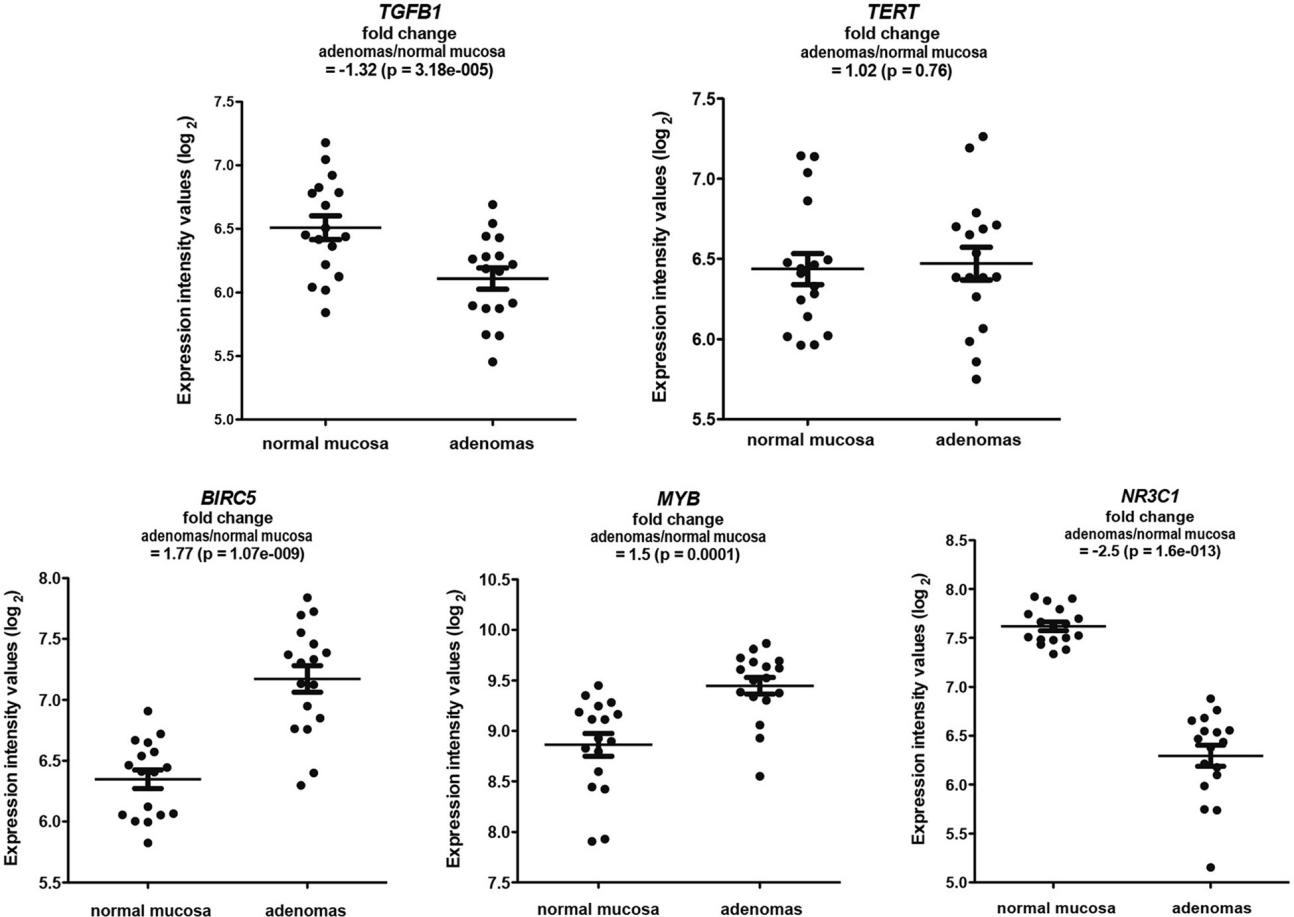
**Transcript levels in colorectal adenomas and normal mucosa for the five target (hub) genes in the TF network shown in Figure **7**.** Scatter plots of normalized log_2_ expression intensity values (y-axis) obtained by Affymetrix Exon 1.0 array analysis of 17 colorectal adenomas and their corresponding samples of normal mucosa. Means and standard errors are represented by horizontal lines and t-bars, respectively. Mean fold changes in adenomas (vs. normal mucosa) are shown for each gene.

For this reason, we used GeneGo (portal.genego.com) to build networks using the 55 TF genes identified by all three of our selection procedures. The most significant network included 27 of the 55 TF genes. Each of the five hub genes identified within this network (*TGFB1*, *BIRC5*, *NR3C1*, MYB, and *TERT*) (Figure 7) is known to play roles in at least one fundamental cellular process involved in tumorigenesis [42-47]. Figure 8 shows how the expression of these hub genes changes as normal colorectal mucosa undergoes adenomatous transformation. The downregulated *TFGB1* transcription we observed in colorectal adenomas (Figure 8) is consistent with previous reports, which described upregulation of this gene only in advanced colorectal tumors (severely dysplastic adenomas and cancers) [48-50]. These findings suggest that the proapoptotic function of *TGFB1*[51], which is important for maintenance of homeostasis in the normal colorectal epithelium, might decline in the early phases of colorectal tumor growth. Indeed, sulindac treatment has been shown to upregulate apoptosis in certain areas of colorectal adenomas, and these same areas also displayed increased *TGFB1* expression [52]. *TGFB1’s* growth inhibition is believed to be replaced by tumor-promoting functions, i.e., immunosuppression and angiogenesis, in more advanced tumors, where its expression is in fact increased [51]. Impaired apoptosis, an essential feature of early adenomatous growth, might also be related to the increased expression of *BIRC5* we documented in our adenomas (Figure 8). *BIRC5* is a well-known member of the inhibitor of apoptosis gene family [45], and its overexpression in precancerous colorectal lesions has been well-documented [53-56].

It is more difficult to predict the functional impact on colorectal tumorigenesis of the striking downregulated expression of the glucocorticoid receptor gene *NR3C1* in all the adenomas we examined (Figure 8). The mechanisms underlying this nuclear receptor’s control of transcription in the intestinal epithelium (and other tissues or cells) are still unknown [57]. Its decreased expression in our adenomas might be related to epigenetic modifications involving its promoter region, which could eventually lead to cytosine hypermethylation as these lesions progress [58]. Upregulated *MYB* expression (Figure 8) has already been reported in human and mouse colorectal tumors, including adenomas [59]. In *APC (Min/+)* mice that are also haploinsufficient for *Myb*, adenoma formation is delayed, and cooperation between Myb and Wnt signaling appears to play a crucial role in this process [60].

As for *TERT*, the fifth hub in this network, its expression in our adenomas was not significantly different from that in normal mucosa (Figure 8). *TERT* is normally expressed in progenitor cells, and its overexpression has been implicated in the transformation of colorectal epithelia and many other types of tumorigenesis as well. Its expression in colorectal adenomas has not been investigated in large studies, but it appears to undergo a gradual increase during progression from adenomas to carcinomas [61,62]. Our adenomas were probably not advanced enough to display significantly upregulated *TERT* expression (all were larger than 10 mm, but most were characterized by low-level dysplasia). Nonetheless, TERT’s putative role as a major player in colorectal cellular transformation emerged from our MetaCore TF analysis, owing in all probability to significant expression changes involving other molecules that interact with TERT in the same network.

In a previous report, we provided a thorough description of the sequential dysregulation of biological pathways that occurs along the adenoma-to-carcinoma sequence, based on analysis of our transcriptomic data [14]. In the present study, we focused on precancerous colorectal lesions and compared our findings with those obtained in colorectal carcinomas using the same approach depicted in Figure 1. Roughly half the TF gene expression perturbations found in carcinomas were already evident in adenomas (Additional file 12: Figure S6; genes listed in Additional file 13: Table S6 and Additional file 14: Table S7), suggesting that the tumorigenic transcriptional program is already well underway during the preinvasive stage. However, a similarly large proportion of TF genes were dysregulated only in carcinomas, which indicates that this program undergoes an important change across the adenoma-to-cancer transition. Extensive validation studies will be necessary to shed light on the biological and clinical implications of the similarities and differences of the transcriptional program between these two stages of transformation.

## Conclusions

This study provides novel information on the TF gene transcript levels associated with adenomatous transformation of the colorectal epithelium and identifies 261 TF genes that appear to play roles in colorectal tumorigenesis. We pinpointed the TF genes whose expression is significantly altered in colorectal adenomas and characterized the extent and direction of these changes. Integrating these findings with those observed in the entire transcriptome allowed us to identify a few hub genes, which may play crucial roles in the formation and progression of adenomas. Finally, we provide useful information on numerous TF genes whose roles in colorectal tumorigenesis have been relatively unexplored, such as *DACH1*, a development gene whose protein expression patterns in colorectal tissues raises interesting questions about its involvement in tumor growth. This study represents a very early step toward a better understanding the highly complex transcription network of a given tissue and tumor. The function of any TF does not depend solely on its expression level but on many other aspects, such as DNA occupancy levels and the tissue-specific availability of factors it interacts with [12,57]. Our findings must be complemented with studies designed to address these aspects of the transcriptional network in colorectal tissues.

## Abbreviations

COBRA: Combined bisulfite restriction analysis; DP: Difference in publications; GO: Gene ontology; MMR: Mismatch repair; NormDP: Normalized DP; TF: Transcription factor; TRANSFAC: TRANScription FACtor.

## Competing interests

All authors deny conflicts of interest relevant to the manuscript.

## Authors’ contributions

JV and MJO performed microarray data analyses and most of the experiments; MM performed COBRA experiments; EC processed microarrays; DP-O histologically classified tissue samples; RH performed immunohistochemistry; JJ conceived important experiments during the study; TS and FB performed endoscopies and tissue sampling; and GM conceived the project, obtained funding, prepared the manuscript, and served as supervising mentor for JV during her Master studies. All authors read and approved the final manuscript.

## Pre-publication history

The pre-publication history for this paper can be accessed here:

http://www.biomedcentral.com/1471-2407/14/46/prepub

## Supplementary Material

Additional file 1: Table S1 Characteristics of the 17 patients with pedunculated adenomas included in the study. Click here for file

Additional file 2: Table S2 Differential expression of 1218 TF genes in colorectal adenomas and corresponding normal mucosa samples. Genes are listed in order of ascending p value; bold-face type: 315 genes with a p value <0.01. Click here for file

Additional file 3: Table S3 Legend of numeric attributes in the MetaCore TF analysis. Click here for file

Additional file 4: Table S4 The 793 TF genes whose networks were enriched in genes displaying significant differential expression in adenomas (Figure 1, MetaCore analysis). Genes are listed in order of descending z-score. Bold-face type: genes with a z-scores > 2; red: "under-researched" genes (NormDP > 0). Click here for file

Additional file 5: Figure S1 Actual and expected numbers of colorectal tumorigenesis-related publications dealing with each TF gene. Relationship between z-score (x-axis) and actual number of publications (y-axis) for each TF gene. The trend line has an intercept = 0 and a slope *alpha* = 142. The *alpha* value can be used to predict the *expected* number of publications. This allowed us to distinguish between TFs that have been "under-researched” (below the trend line) and "over-researched“ (above the trend line) in the field of colorectal tumorigenesis. Click here for file

Additional file 6: Table S5 Characteristics of the 80 colon cancer patients tested for DACH1 expression. Click here for file

Additional file 7: Figure S2 Hierarchical clustering analysis of colorectal tissue samples based on expression levels of 1218 TF genes. (Pearson correlation, Ward distance). The 34 tissue samples represented on the *x*-axis include 17 normal mucosal samples (normal) and 17 adenomas (adenoma). Each transcript probe set plotted on the *y*-axis is color-coded to reflect expression levels of the 1218 TF genes relative to their median expression levels across the entire tissue-sample set (red: high; green: low). Click here for file

Additional file 8: Table S8 Scores of 261 TF genes that passed at least 2 of the 3 selection thresholds shown in Figure 1. Genes are listed in the order of ascending p values. Click here for file

Additional file 9: Figure S3 DACH1 immunohistochemical staining of sections from formalin-fixed, paraffin-embedded pellets of 6 colon cancer cell lines. Affymetrix U133Plus2.0 raw mRNA *DACH1* expression values are reported for each cell line. These gene expression levels were consistent with the complete absence of DACH1 protein in CO115 cells (A) and its extremely weak expression in HCT116 cells (B). Strongly stained nuclei were rare in the population of Caco2 cells (C) but much more common in LS180 cells (D). Most of the SW620 cell nuclei were positive for DACH1 expression, which is consistent with the high *DACH1* mRNA expression value for these cells (E). Interestingly, DACH1 expression was absent in SW480 cells (F), which were established from a primary colon cancer whose lymph node metastasis was used to establish the SW620 cell line. Click here for file

Additional file 10: Figure S4 Legend of the symbolic attributes of the gene networks shown in Figure 7 and Additional file 11: Figure S5. Click here for file

Additional file 11: Figure S5 The most significant network included 27 of the 55 TF genes found in all three sets depicted in Figure 2. In this panel, the *subcellular localization* layout of MetaCore is shown. Click here for file

Additional file 12: Figure S6 Venn diagrams showing intersection of TF gene sets identified in our analyses of colorectal *adenomas* and *carcinomas* (each compared with matched samples of normal mucosa). Top diagram: The number of TF genes was identified with the *t* test-based procedure shown in Figure 1 (left prong). Bottom diagram: The number of TF genes was identified with the MetaCore-based procedure shown in Figure 1 (right prong). Note that the number of TF genes identified with the latter approach in adenomas and carcinomas refer to version 6.16, build 63671 of MetaCore™ software. Click here for file

Additional file 13: Table S6 Differential expression of 1038 TF genes in colorectal *carcinomas* vs. corresponding samples of normal mucosa. Genes are listed in order of ascending p values. Bold-face type: 232 genes with a p value <0.01. The 107 TF genes listed in red were also dysregulated in adenomas (see Venn diagram in Additional file 11: Figure S5). Click here for file

Additional file 14: Table S7 The 633 TF genes whose networks were enriched in genes displaying significant differential expression in *carcinomas* (MetaCore analysis). Genes are listed in order of descending z-scores. The 255 genes listed in bold-face type had z-scores of > 2. Click here for file
