# Supplementary material for: A comprehensive look at transcription factor gene expression changes in colorectal adenomas
Source: BMC Cancer. 2014 Jan 29;14:46. doi: 10.1186/1471-2407-14-46 (PMC4078005; doi:10.1186/1471-2407-14-46)
Supplement: Additional file 3: Table S3 — Legend of numeric attributes in the MetaCore TF analysis. [file 1471-2407-14-46-S3.pdf]

Supplementary Table 3: Legend of numeric attributes in the MetaCore TF analysis

**Object name**	network object name in MetaBase
**Actual**	number of targets in the activated dataset(s) regulated by the chosen transcription factor (TF)
**n**	number of network objects in the activated dataset(s)
**R**	number of targets in the complete database or background list regulated by the chosen TF
**N**	total number of gene-based objects in the complete database or background list
**Expected**	mean value for hypergeometric distribution (n*R/N)
**Ratio**	connectivity ratio (Actual/Expected)
**z-score**	z-score ((Actual-Expected)/sqrt(variance))
**p-value**	probability to have the given value of Actual or higher (or lower for negative z-score)
