# Supplementary figures and images for: A comprehensive look at transcription factor gene expression changes in colorectal adenomas
Source: BMC Cancer. 2014 Jan 29;14:46. doi: 10.1186/1471-2407-14-46 (PMC4078005; doi:10.1186/1471-2407-14-46)

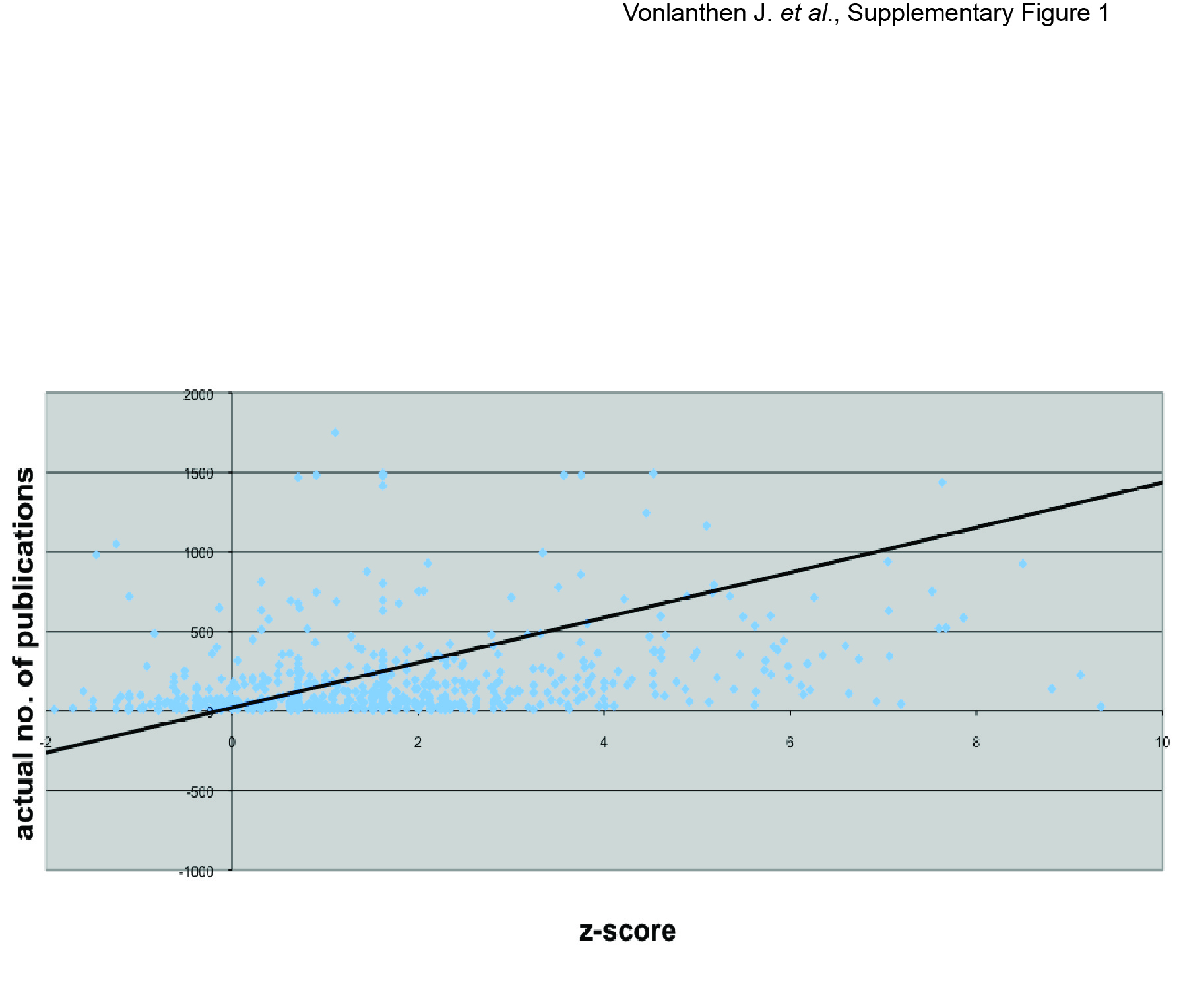

Supplement: Additional file 5: Figure S1 — Actual and expected numbers of colorectal tumorigenesis-related publications dealing with each TF gene. Relationship between z-score (x-axis) and actual number of publications (y-axis) for each TF gene. The trend line has an intercept = 0 and a slope alpha = 142. The alpha value can be used to predict the expected number of publications. This allowed us to distinguish between TFs that have been "under-researched” (below the trend line) and "over-researched“ (above the trend line) in the field of colorectal tumorigenesis. [file 1471-2407-14-46-S5.jpeg]

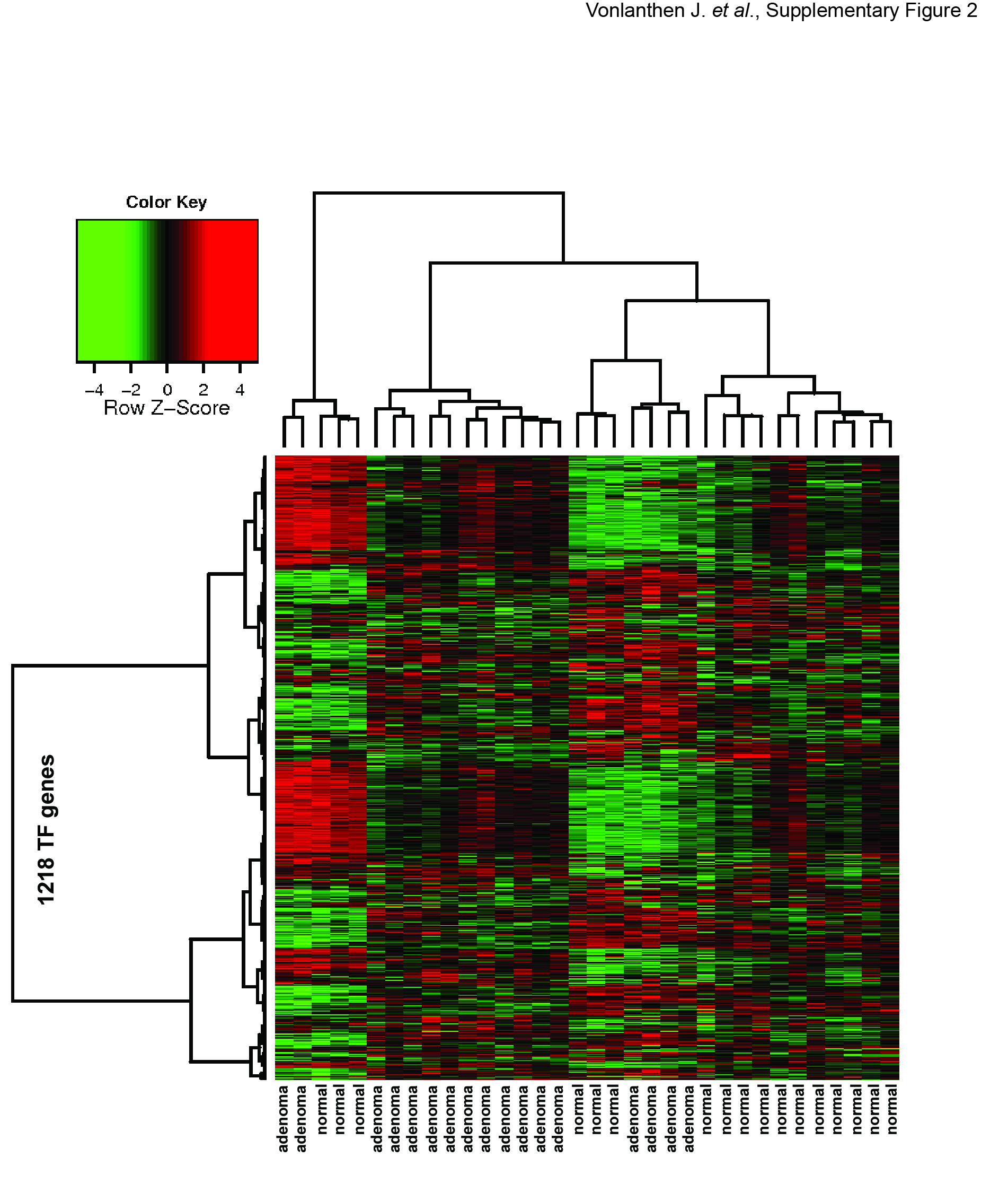

Supplement: Additional file 7: Figure S2 — Hierarchical clustering analysis of colorectal tissue samples based on expression levels of 1218 TF genes. (Pearson correlation, Ward distance). The 34 tissue samples represented on the x-axis include 17 normal mucosal samples (normal) and 17 adenomas (adenoma). Each transcript probe set plotted on the y-axis is color-coded to reflect expression levels of the 1218 TF genes relative to their median expression levels across the entire tissue-sample set (red: high; green: low). [file 1471-2407-14-46-S7.jpeg]

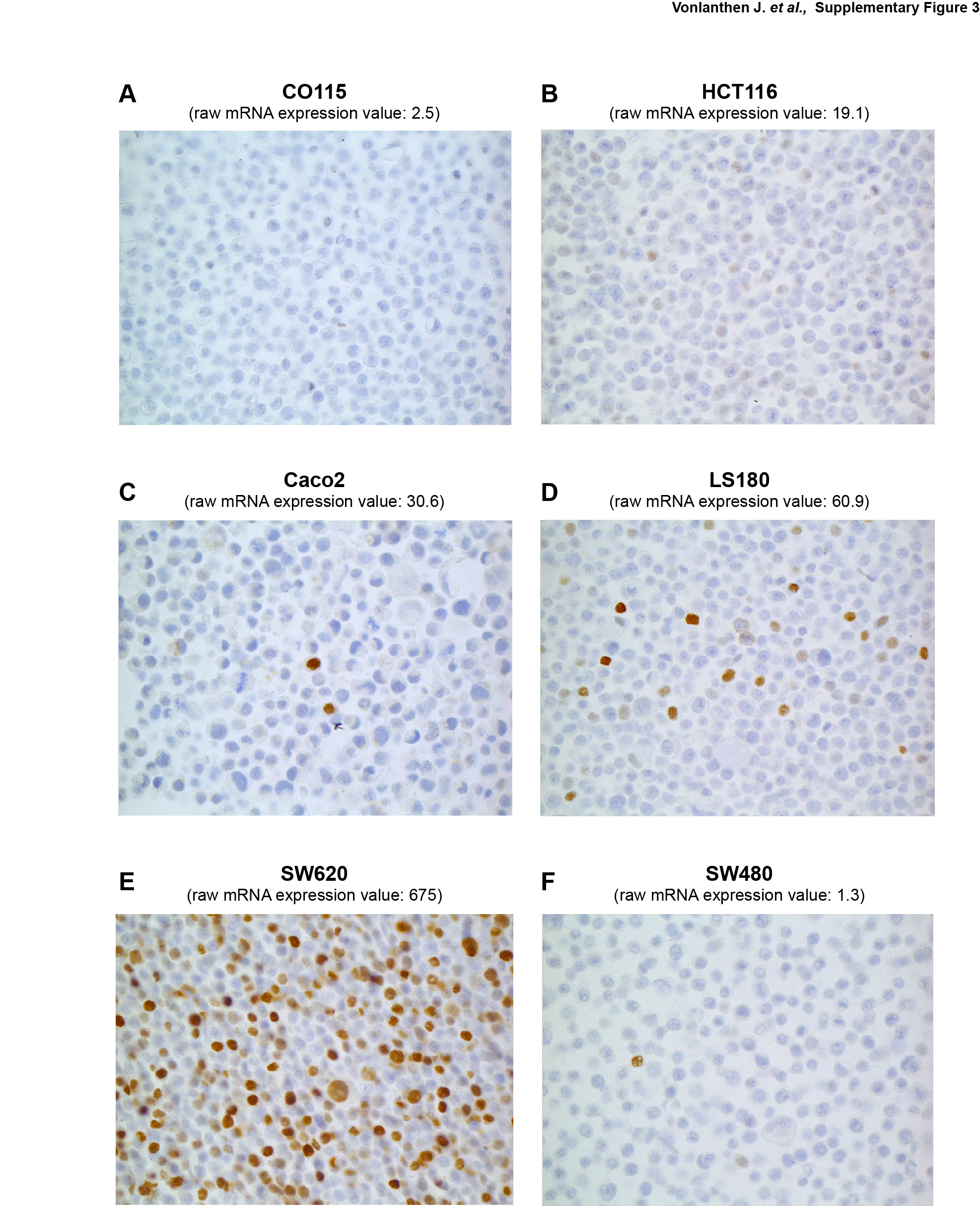

Supplement: Additional file 9: Figure S3 — DACH1 immunohistochemical staining of sections from formalin-fixed, paraffin-embedded pellets of 6 colon cancer cell lines. Affymetrix U133Plus2.0 raw mRNA DACH1 expression values are reported for each cell line. These gene expression levels were consistent with the complete absence of DACH1 protein in CO115 cells (A) and its extremely weak expression in HCT116 cells (B). Strongly stained nuclei were rare in the population of Caco2 cells (C) but much more common in LS180 cells (D). Most of the SW620 cell nuclei were positive for DACH1 expression, which is consistent with the high DACH1 mRNA expression value for these cells (E). Interestingly, DACH1 expression was absent in SW480 cells (F), which were established from a primary colon cancer whose lymph node metastasis was used to establish the SW620 cell line. [file 1471-2407-14-46-S9.jpeg]

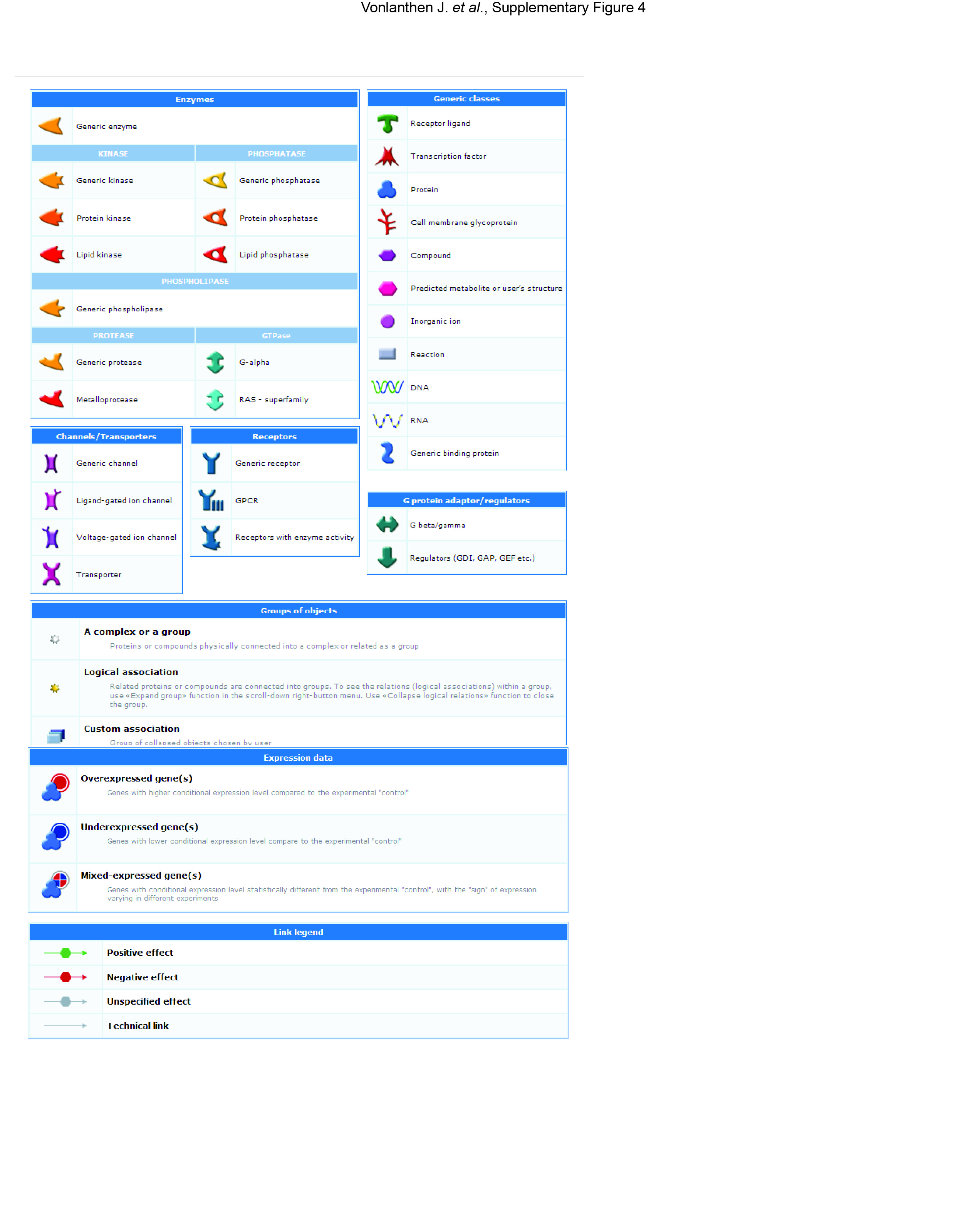

Supplement: Additional file 10: Figure S4 — Legend of the symbolic attributes of the gene networks shown in Figure 7 and Additional file 11: Figure S5. [file 1471-2407-14-46-S10.jpeg]

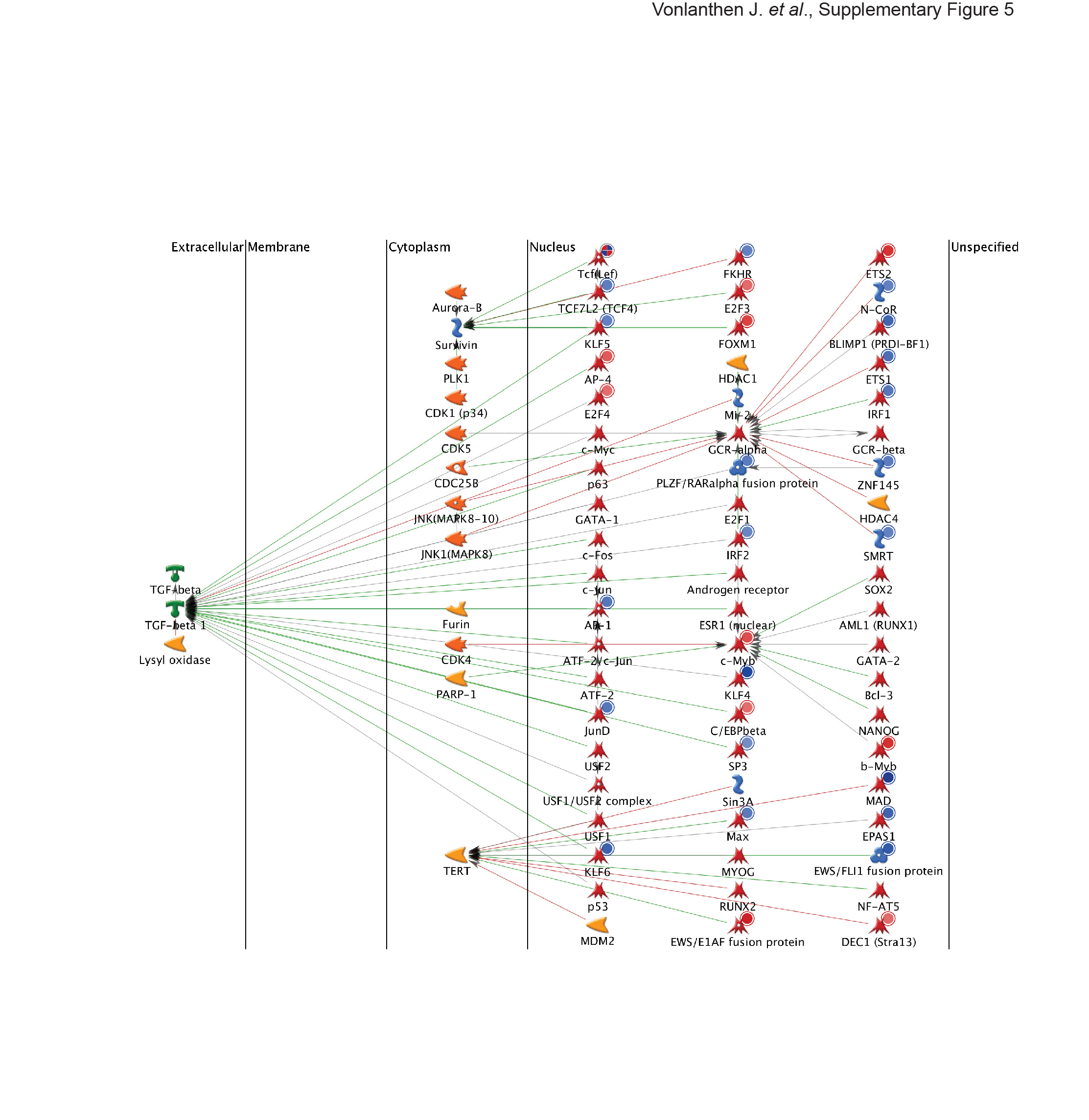

Supplement: Additional file 11: Figure S5 — The most significant network included 27 of the 55 TF genes found in all three sets depicted in Figure 2. In this panel, the subcellular localization layout of MetaCore is shown. [file 1471-2407-14-46-S11.jpeg]

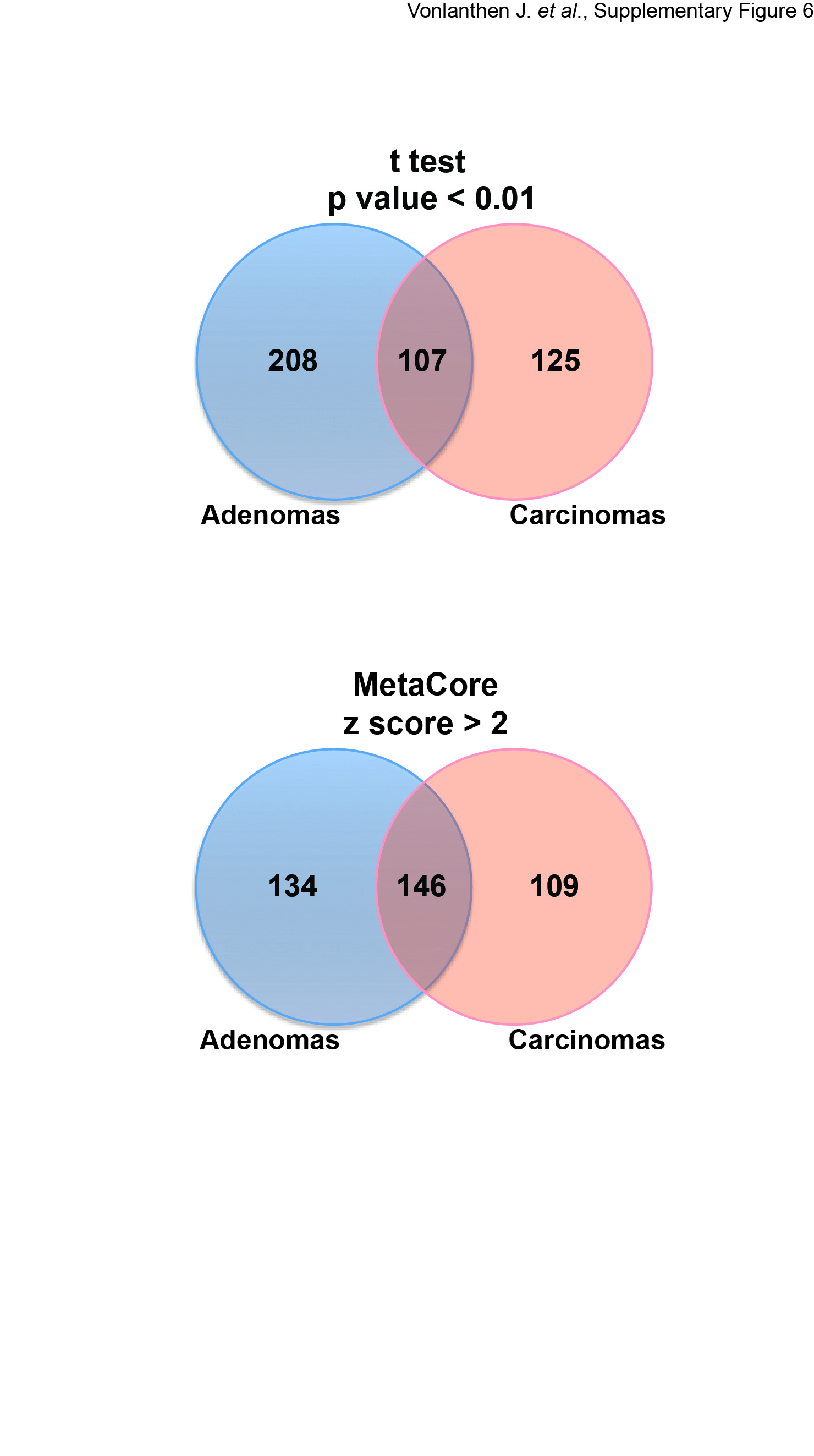

Supplement: Additional file 12: Figure S6 — Venn diagrams showing intersection of TF gene sets identified in our analyses of colorectal adenomas and carcinomas (each compared with matched samples of normal mucosa). Top diagram: The number of TF genes was identified with the t test-based procedure shown in Figure 1 (left prong). Bottom diagram: The number of TF genes was identified with the MetaCore-based procedure shown in Figure 1 (right prong). Note that the number of TF genes identified with the latter approach in adenomas and carcinomas refer to version 6.16, build 63671 of MetaCore™ software. [file 1471-2407-14-46-S12.jpeg]
